# Tracking personalized functional health in older adults using geriatric assessments

**DOI:** 10.1186/s12911-020-01283-y

**Published:** 2020-10-20

**Authors:** Anup K. Mishra, Marjorie Skubic, Mihail Popescu, Kari Lane, Marilyn Rantz, Laurel A. Despins, Carmen Abbott, James Keller, Erin L. Robinson, Steve Miller

**Affiliations:** 1 Department of Electrical Engineering and Computer Science, University of Missouri, Columbia, MO 65211 USA; 2 Department of Health Management and Informatics, University of Missouri, Columbia, MO 65211 USA; 3 Sinclair School of Nursing, University of Missouri, Columbia, MO 65211 USA; 4 School of Health Professions, Physical Therapy, University of Missouri, Columbia, MO 65211 USA; 5 School of Social Work, University of Missouri, Columbia, MO 65211 USA

**Keywords:** Functional health, Geriatric assessments, Older adults, Personalized functional health trajectory, Health status indicators, Mixed effects modeling

## Abstract

**Background:**

Higher levels of functional health in older adults leads to higher quality of life and improves the ability to age-in-place. Tracking functional health objectively could help clinicians to make decisions for interventions in case of health deterioration. Even though several geriatric assessments capture several aspects of functional health, there is limited research in longitudinally tracking personalized functional health of older adults using a combination of these assessments.

**Methods:**

We used geriatric assessment data collected from 150 older adults to develop and validate a functional health prediction model based on risks associated with falls, hospitalizations, emergency visits, and death. We used mixed effects logistic regression to construct the model. The geriatric assessments included were Activities of Daily Living (ADL), Instrumental Activities of Daily Living (IADL), Mini-Mental State Examination (MMSE), Geriatric Depression Scale (GDS), and Short Form 12 (SF12). Construct validators such as fall risks associated with model predictions, and case studies with functional health trajectories were used to validate the model.

**Results:**

The model is shown to separate samples with and without adverse health event outcomes with an area under the receiver operating characteristic curve (AUC) of > 0.85. The model could predict emergency visit or hospitalization with an AUC of 0.72 (95% CI 0.65–0.79), fall with an AUC of 0.86 (95% CI 0.83–0.89), fall with hospitalization with an AUC of 0.89 (95% CI 0.85–0.92), and mortality with an AUC of 0.93 (95% CI 0.88–0.97). Multiple comparisons of means using Turkey HSD test show that model prediction means for samples with no adverse health events versus samples with fall, hospitalization, and death were statistically significant (p < 0.001). Case studies for individual residents using predicted functional health trajectories show that changes in model predictions over time correspond to critical health changes in older adults.

**Conclusions:**

The personalized functional health tracking may provide clinicians with a longitudinal view of overall functional health in older adults to help address the early detection of deterioration trends and decide appropriate interventions. It can also help older adults and family members take proactive steps to improve functional health.

## Background

The number of Americans ages 65 and older is projected to be over 98 million by 2060, which is about 24 percent of the total population in the USA [1]. The aging population is at a higher risk of functional decline than their younger counterparts [2]. Keeping older adults at higher functional levels can lead to higher quality of life, successful aging-in-place, and reduce healthcare expenditures [3]. Hence, continuous tracking of functional health (FH) is necessary.

FH in older adults is complex and multifactorial [4, 5]. Gordon has defined 11 FH patterns to facilitate nursing diagnoses [4]. The list of FH patterns included health-perception, activities of daily living, cognitive ability, and self-perception. This suggests that FH is not only limited to physical function, but rather is a combination of physical, cognitive, and social function, among other factors. The World Health Organization’s 2015 World Report on Aging and Health outlines a framework for Aging-in-Place around the new concept of functional ability [5]. It reinforces that FH is a combination of physical, cognitive, and social function, and also suggests that the loss of these functions has a detrimental impact on an older adult’s health status, quality of life, and independence [5, 6]. Therefore, in this study, we have used a specific set of geriatric assessments that can measure multiple aspects of physical, cognitive, and social function to predict overall FH.

Geriatric assessments such as Activities of Daily Living (ADL), Instrumental Activities of Daily Living (IADL), Mini-Mental State Examination (MMSE), Geriatric Depression Scale (GDS), and Short Form 12 (SF12) measure multiple aspects of FH in older adults [7–16]. Previous studies suggest that using a combination of health assessments could be effective in predicting health status and outcomes [17, 18]. Therefore, instead of using these individual assessments for health monitoring, an integration of these assessments could be used to track FH more effectively. Also, reducing the number of measures to track health could support the monitoring responsibility of health care professionals by saving time and facilitating early functional decline detection.

Several studies have been conducted in developing health and prognostic indexes to track or predict multimorbidity, mortality, frailty, and physical FH in older adults [17, 19–23]. Mazzaglia et al. developed two prognostic index models to predict 5-month mortality and hospitalization [17]. In the first model, they used a set of 7 questions from ADL and IADL to develop their index. The area under the receiver operating characteristic curves (AUC) to predict mortality and hospitalizations were 0.75 and 0.60, respectively. In the second model, they considered drug use and previous hospitalizations, which increased their hospitalization AUC to 0.67. Gagne et al. developed a single numeric index to predict mortality by combining Charlson and Elixhauser measures [19]. Results show that the combined score performed better in predicting mortality than the individual scores. Carey et al. and Lee et al. developed prognostic models to predict mortality using data from the Program of All‐Inclusive Care for the Elderly (PACE) and 1998 wave of the Health and Retirement Study (HRS), respectively [20, 21]. Schonberg et al. used 39 risk factors, including functional measures, illnesses, behaviors, demographics in a multivariable Cox proportional hazards model to predict 5-year mortality [22]. Giovanni et al. developed a multisource comorbidity score using administrative data, such as diagnostic categories and ICD-9 to measure comorbidity, predict 1-year mortality, and other adverse outcomes [23]. The study did not include functional status as a variable in the predictive model development.

Fried et al. conducted a study to predict frailty in older adults [24]. They defined frailty as a clinical syndrome in which the older adult has three or more out of five frailty criteria. These five criteria include unintentional weight loss, self-reported exhaustion, weakness, slow walking speed, and low physical activity. This standardized phenotype of frailty detection can identify frail older adults potentially at risk of falls, hospitalizations, disability, and death. Rockwood et al. developed a 7-point Clinical Frailty scale to predict death or need for institutional care [25]. The Clinical Frailty scale is based on an a-priori selection of features and is intended to predict mortality or need for institutional care.

The mortality-based prognostic models tend to predict future adverse health conditions, specifically death, instead of predicting the overall FH of an individual at a given time. The frailty phenotyping method developed by Fried et al. can predict the presence of frailty with minimal granularity as it can only classify an older adult into one of the three frail categories: frail, intermediate frail, and not frail [24]. This may not be able to track the gradual changes in the FH of an individual. Also, this only considers the physical aspects of FH; cognitive aspects were excluded [26, 27]. We argue that a continuous measure of overall FH can provide critical information about changes in FH over time. Interventions based on overall FH deterioration can help older adults live with higher independence and quality of life [28].

Santoni et al. used gait speed, cognitive function, chronic multimorbidity, and disability to predict present and future care needs in Swedish older adults [18]. Their model could predict hospitalization with an AUC of 0.78 (95 CI = 0.74–0.81) and mortality with an AUC of 0.85 (95% CI = 0.83–0.87). The dataset used in the study included older adults with high levels of cognitive and physical function; at least 90% of participants were free of severe disability, and at least 50% were functionally independent despite chronic disorders. In contrast, in our study, the dataset includes older adults with comparatively lower cognitive and physical function. The study did not include falls and emergency visits as outcome measures.

We hypothesized that detecting a decline in an individual’s FH would represent deterioration in underlying health conditions recorded in the electronic health record. In this study, we develop and validate a method for continuous tracking of personalized FH of older adults using routine geriatric assessments and adverse health outcomes. We use a mixed effects logistic regression model that allows us to use repeated measurements to build the model and provide personalized health predictions. We hypothesize that these geriatric assessments would provide sufficient information in developing a personalized FH tracking model. We believe that continuous tracking of FH could help early detection of health deteriorations and facilitate earlier interventions by health professionals to improve the health of an older adult.

## Methods

### Data

The proposed model of the personalized FH is based on a set of frequently collected geriatric assessment scores in the Electronic Medical Record (EMR), such as ADL (Short Form ADL, RAI MDS 2.0), IADL (Lawton), GDS, MMSE, and SF12 [27, 29, 30]. The SF-12 assessment has two components, a physical component or PCS and a mental component or MCS. We used assessments routinely collected at TigerPlace, an Aging-in-Place facility in Columbia, MO, on 150 independent living older adult residents (females = 97, age = 87.2 ± 7.2) [31]. The assessments were obtained by the nursing staff working at TigerPlace in collaboration with the Sinclair Nursing School at the University of Missouri, Columbia. All assessments were collected at an interval of approximately six months. The assessment data included were collected over eight years, specifically from 2011 to 2019. The dataset had 12.2% missing assessments. Missing assessment scores in the final dataset were imputed by using the most recent assessment scores. Multi-collinearity was determined using the Pearson correlation coefficient for the assessments. None of the included assessment pairs had a Pearson correlation greater than 0.7. The final dataset contained 4,495 individual assessments. The number of assessments in each assessment category were comparable. Table 1 shows a summary of the characteristics of the assessment data.

**Table 1:** Assessment data characteristics

Assessments (range)*	Mean (Std)
ADL (0–16)	2.19 (3.23)
IADL (0–8)	3.88 (1.57)
MMSE (0–30)	25.09 (6.54)
GDS (0–15)	2.88 (2.45)
SF-12, mental score (0–100)	54.31 (9.17)
SF-12, physical score (0–100)	37.76 (11.85)

^*^Interpretation of the assessment scores—ADL, higher scores indicate more ADL impairment; IADL, lower scores show low function; MMSE, lower scores show more cognitive impairment; GDS, higher scores indicate depression; SF-12, low scores indicate low level of mental or physical health

In developing the model, samples with any of the four adverse health outcome categories including falls, emergency visits, hospitalizations, and death were considered to be the positive class. The emergency visits included in this study are only emergency department visits, excluding urgent care and physician office visits. These health events were assumed to reflect the underlying FH deteriorations of an individual. The dataset contained 2,677 health events, out of which 1,931 were falls. The health events were reported by the TigerPlace staff in the EMR.

This study received Institutional Review Board approval at the University of Missouri, Columbia.

### Model construction

In the model construction, samples corresponding to the adverse health events of fall, hospitalization, emergency visits, and death were considered as the positive class, and rest were considered as the negative class. Samples are a set of five assessments (ADL, IADL, MMSE, GDS, SF-12) collected together at a given six-month period for a resident. Adverse health events associated with the study participants were overlapped. Therefore, instead of considering them as individual classes, we considered all samples with any number of adverse health events as one class for the model development.

We used mixed effects logistic regression to develop the model using repeated assessment data from the residents [32, 33]. Mixed effects logistic regression is a type of generalized linear mixed model (GLMMs). This model is used to model binary outcome variables, in which the log odds of the outcomes are modelled as a linear combination of predictor variables, specifically when there are both fixed and random effects in the data [33]. The general form of the GLMM model is,

1 $$\mathbf{y}=h({\varvec{\eta}})+{\varvec{\varepsilon}}$$

where $\mathbf{y}$ is a column matrix of the outcome variable, and $h(\cdot )$ is the inverse link function. In this study, we used the logistic inverse link function to build the model. The logistic inverse link function can be represented as,

2 $$h(\cdot )=\frac{{e}^{(\cdot )}}{1+{e}^{(\cdot )}}$$

${\varvec{\eta}}$ is the linear predictor, which can be represented as the combination of fixed and random effects as shown in Eq. (3).

3 $${\varvec{\eta}}={\varvec{X}}{\varvec{\beta}}+{\varvec{Z}}{\varvec{\gamma}}$$

${\varvec{X}}$ is the matrix of predictor variables, ${\varvec{\beta}}$ is a column vector of the fixed-effect regression coefficients, ${\varvec{Z}}$ is the design matrix for the random effects, ${\varvec{\gamma}}$ is the vector of random effects, and ${\varvec{\varepsilon}}$ is the column vector of residuals [34].

In this study, the assessments collected over time are nested within the residents. The assessments were considered as fixed effects, and the residents were considered as the random effects because assessment measures collected within the residents may be correlated. Modelling residents as a random effect provides the personalization effect, as the model predictions depend on who the resident is instead of just the assessment scores. The predicted probabilities from the final model were subtracted from 1.0 so that higher values represent better health status and vice versa. We refer to these values as functional health values (FHV) in the rest of the article.

### Model assessment and construct validators

The model is not designed to predict a specific condition, instead, it is designed to predict any of the four adverse health events. To validate the model, we followed the construct validity methodology adopted by Richardson et al. in developing the Score for Neonatal Acute Physiology, respectively [35]. Boudreaux et al. defined construct validity as ‘‘... the degree to which a measure actually assesses the attribute it is purported to measure’’ based on ‘‘whether the measures relate to other variables in expected and predictable ways’’ [36, p. 168]. The relationship of the FHV to a health event category independently associated with FH was examined on the assumption that declining FH is expected to correspond to more detrimental health events.

Three construct validators were chosen to validate the model. Validation results are reported using sensitivity–specificity analysis and area under the receiver operating characteristic curves (AUC) [37, 38]. The correspondence of FHV to different health events was evaluated. The five health event categories considered for this evaluation were: no adverse health event, hospitalization or emergency visit only, fall only, fall with hospitalizations, and death only. Fall with hospitalizations category corresponds to samples with falls that cause hospitalizations. The rest of the categories had independent samples without any overlap with other categories.

#### Health event categories versus FHV

FHV for all health event categories were computed. We employed analysis of variance (ANOVA) procedures to determine the statistical significance between FHV associated with the different health categories. The ANOVA analysis was followed with multiple comparisons of means using the Turkey HSD post-hoc test [39]. Also, the FHV were used to separate different health event categories, specifically, no health events versus the rest. The area under the curve (AUC) associated with separating no health event category with rest was calculated.

#### Six-month fall

Falls represented the largest category of adverse health events in our data set. Therefore, to validate the effectiveness of FHV in predicting a fall within six months, the average six-month fall percentages associated with FHV were computed. We considered samples from both falls only and falls with hospitalization categories for this analysis.

#### Case studies

The basic idea of developing the FH prediction model is to track the personalized FH of older adult residents. Also, we wanted to evaluate if the changes observed in the FH trajectories correspond to the underlying health condition of the residents. Therefore, we explored two case studies to evaluate the correspondence of observed changes in FH trajectories with actual FH changes documented in EMR. FHV were computed for the entire stay of these residents at TigerPlace. A timeline of FHV was plotted to represent the FH trajectory of each resident. An investigation of the clinical notes was performed to obtain the actual FH changes reported in the EMR for these residents. The ground truth on FH changes reported in clinical notes were compared with the changes observed in the FH trajectory. See “Appendix 1” for more examples of FH trajectories along with a timeline representation of adverse health events for the residents.

## Results

In this section, we present the results associated with each construct validator.

### Health event categories versus FHV

Table 2 shows the mean, standard deviation, and sample size of data in each health event category. Mean values for the five categories show that FHV associated with no health events were higher when compared to the samples associated with hospitalization, emergency visit, fall, and death.

**Table 2:** Mean FHV by health event category

Health event category	FHV (n = 899) Mean (Std), Sample size
No health event	0.69 (0.18), 497
Emergency visit or hospitalization only	0.54 (0.18), 55
Fall only	0.38 (0.07), 224
Fall with hospitalization	0.34 (0.20), 92
Death*	0.30 (0.16), 31

^*^Six samples with death events were overlapped with fall with hospitalization. These samples were considered under the Death event category and excluded from the Fall and hospitalization category

**Fig. 1:**
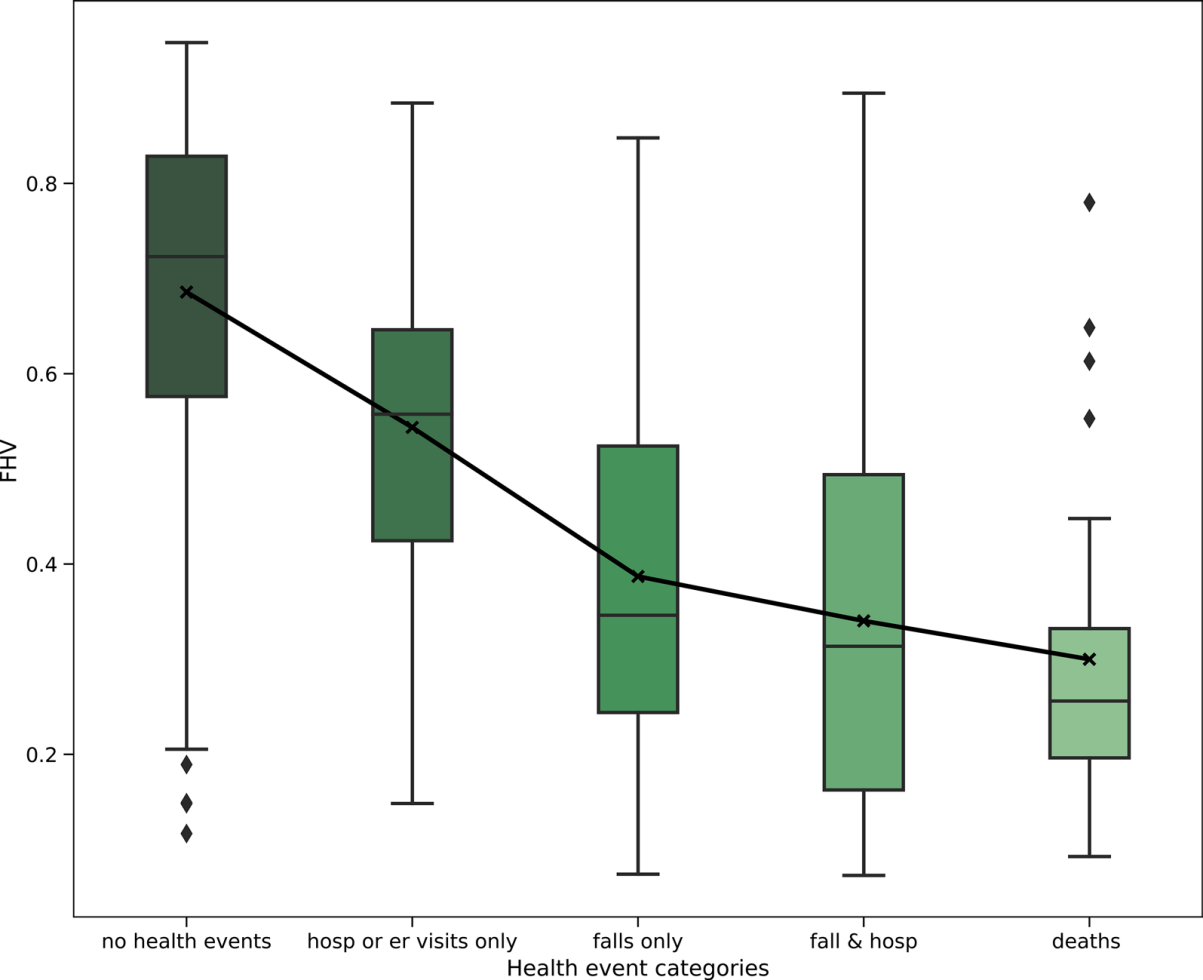
FHV versus health event categories. The top and bottom of each box represent 75% and 25% percentiles of FHV for that category. Horizontal lines in each box represent the median FHV values for each category. The top of each whisker represents the maximum FHV in that category or median plus 1.5 times the interquartile range; the bottom whisker represents the minimum FHV in that category or median minus 1.5 times the interquartile range

**Fig. 2:**
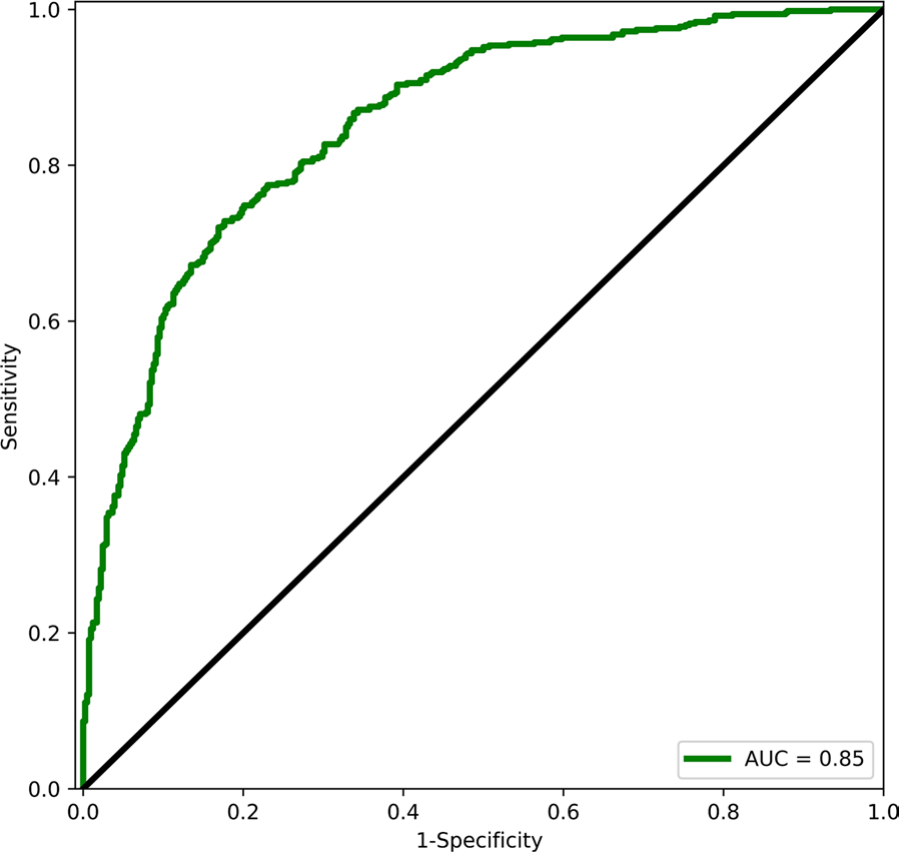
Receiver operating curve showing the separation of the no health event category from the rest (emergency visit/hospitalization, fall, fall and hospitalization, and death)

A one-way ANOVA was calculated on FHV values associated with the different health event categories. The analysis was significant (*F* = 154.99, *p* < 0.0001). Multiple comparisons of means using the Turkey HSD test show that all pairs of health event categories were statistically significant (p < 0.001), except for two pairs: fall only versus fall with hospitalization and death versus fall with hospitalization [40]. Figure 1 shows FHV for different health event categories. FHV for no health events were well separable from the rest of the health events with an AUC value of 0.85 (95% CI 0.83–0.88). Figure 2 shows the receiver operating curve for the separation.

The model could predict emergency visit or hospitalization with an AUC of 0.72 (95% CI 0.65–0.79), fall only with an AUC of 0.86 (95% CI 0.83–0.89), fall with hospitalization with an AUC of 0.89 (95% CI 0.85–0.92), and death with an AUC of 0.93 (95% CI 0.88–0.97) when separating from no health event category. Additional file 1: Table 1 shows the predictive ability of FHV compared to the individual health assessments when separating no health events category from the rest of the adverse health event categories.

### Six-month fall

**Fig. 3:**
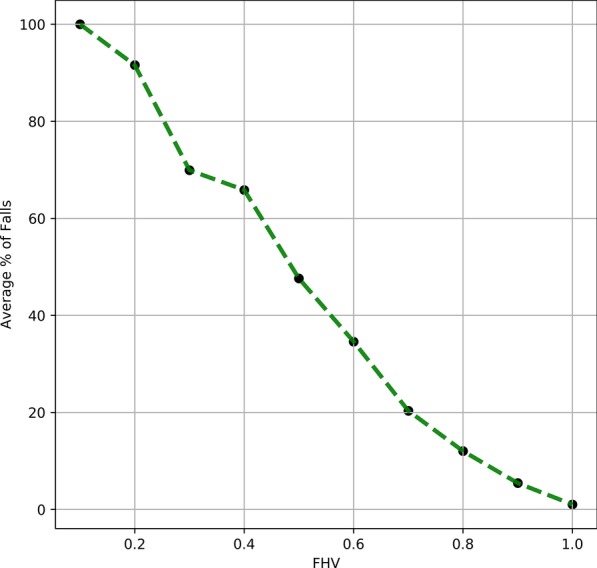
Average % of Six-Month Falls versus FHV

The average likelihood of fall within six months of computing the FHV is shown in Fig. 3. We observed that higher FHV correspond to a lower average fall percentage and vice versa. An FHV score of 1.0 corresponds to ~ 0.0 fall percentage. The fall percentage almost linearly increased with decrease in FHV.

### Case studies

Two case studies are presented demonstrating correspondence of changes in FHV with significant health changes reported in EMR nursing notes.

#### Case study-1

**Fig. 4:**
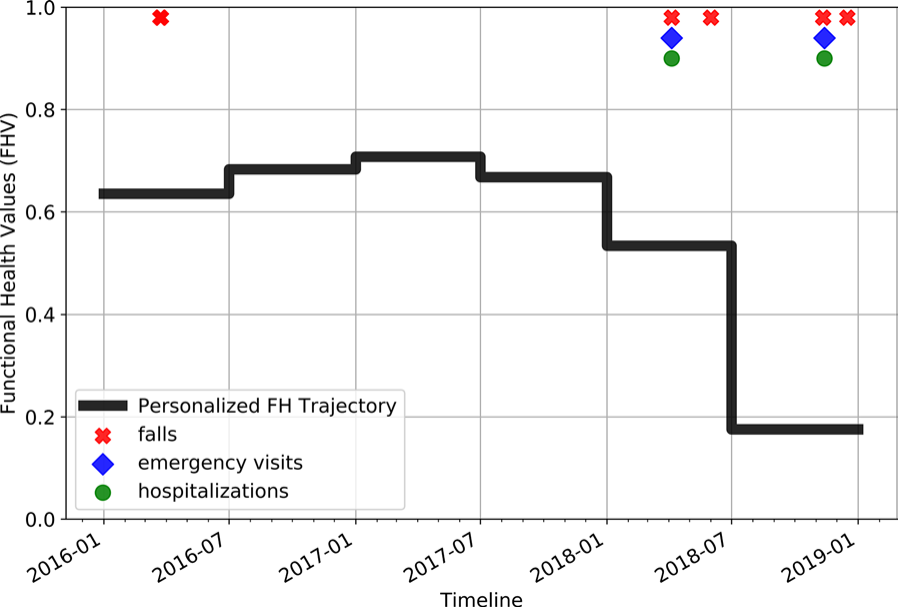
FH trajectory of a TigerPlace resident from Case Study-1

Figure 4 shows the FH trajectory of a TigerPlace resident. Falls, emergency visits, and hospitalizations experienced by the resident are marked on the FH trajectory timeline. A visual assessment of the plot suggests that lower FHV correspond to falls, emergency visits, and hospitalizations. The resident did not experience any critical health event during the period between July 2016 to January 2018 when the FHV were higher.

An analysis of EMR clinical notes suggests that the resident was able to walk independently without any support until January of 2018. The resident started walking with a walker and was often in a wheelchair starting from April 2018. A sharp decline in the FHI trajectory at the beginning of 2018, with FHV < 0.6, confirms that FHV decline may correspond to significant FH changes.

#### Case Study-2

**Fig. 5:**
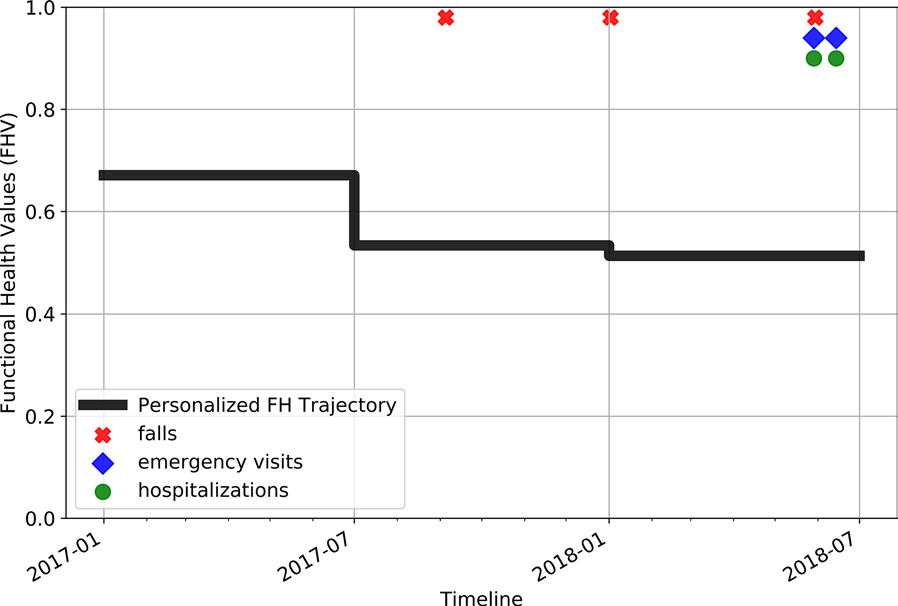
FH trajectory of a TigerPlace resident from Case Study-2

Figure 5 shows the FH trajectory of another resident at TigerPlace. An investigation of the resident’s EMR clinical notes suggests that the resident had chronic pain and was in a wheelchair for the entire period.

Also, EMR notes show that the resident had a fall in September 2017, experienced increased back pain during October 2017, and cognitive impairment during November 2017. These health changes correspond to the significant decline in FHV observed in the second half of 2017. FHV for the resident further declined in the later months (FHV ~ 0.5) corresponding to further FH deterioration, leading to falls, emergency visits, and hospitalizations between 01–2018 and 07–2018. This shows that a decline in FHV may correspond to cognitive impairments and other FH deteriorations.

## Discussion

We have developed an FH prediction model to track continuous personalized FH using a set of geriatric assessments. An FH trajectory is developed using longitudinal FHV predictions over time. The FH trajectory can be updated for a resident as new assessment scores are available, typically every six months. The model was developed by discriminating geriatric assessment scores associated with adverse health events, such as falls, emergency visits or hospitalizations, and mortality against scores associated with no adverse health events.

Results show that a rank order was observed in mean FHV, when moving from a lower health risk category, such as no health events to a higher health risk category, such as fall with hospitalization and death. This shows the generality of FHV. FHV can be interpreted to the effect that a higher value represents a healthier person. Results show that FHV of > = 0.6 corresponds to < 40% of six-month fall risk. The six-month fall risk percentage almost linearly increases with a decrease in FHV. Results also show that FHV < 0.4 could significantly increase the risk of falls, hospitalizations, and mortality. Case study analyses suggest that changes in FH trajectories are mostly gradual with some sudden drops. Sudden drops in FHV did correspond to significant health changes observed in the EMR. A lower FHV, specifically FHV < 0.4 throughout could suggest a high risk of falls and hospitalizations for the entire stay of the older adult. See “Appendix 1” for more case studies and FH trajectory plots.

The high AUC values of 0.85 obtained for separating samples corresponding to no health events from rest suggests that a higher value of FHV represent a healthier FH state of the resident. FHV is not intended to predict a specific event. Instead, FH trajectories over a period can show the trend of FH changes for an individual. The case studies discussed above show that change in FHV may indicate a change in physical or cognitive FH. A decline in FHV below 0.6 may indicate a severe FH decline and interventions are needed to improve or maintain the FH of the resident. In the case studies, we observed that FHV below 0.6 was associated with an increased number of falls, hospitalizations, and emergency visits. In the case of the second case study, the FH trajectory shows that FHV of the resident moved below 0.6 in the latter half of 2017. However, the person started experiencing adverse health events in early 2018. We believe that early interventions, specifically in the second case study may have helped the resident to possibly improve overall FH and avoid the following health events.

In [31], Rantz et al. conceptualized that the functional ability tends to decline unless timely interventions are provided. As we studied the FH trajectories for the TigerPlace residents, we found that the FH of an individual can decline as well as improve. We observed that for some residents, as they first start living at TigerPlace, their FH improved over a period. This could be because of the state-of-the-art care coordination provided at TigerPlace and other similar facilities. We observed this effect in the first case study. The predicted FH of the resident improved between 2016 and 2017 before it started to decline in the last half of 2017.

A limitation of this study is the study sample size. We had access to the data of only 150 senior residents from a single aging-in-place facility. We believe that data from a larger population, with more assessments and health events could improve the generalizability of the model.

A second limitation is that we did not incorporate multimorbidity or age in our model. Previous studies have included chronic morbidity was as number of chronic conditions to predict health outcomes [18]. While age and multimorbidity are associated with increased adverse health events, we were interested in evaluating the effectiveness of a composite score based on the routinely obtained geriatric assessments reported in the EMR in detecting health changes.

The use of mixed effects modeling to predict adverse health events from repeated measurements from the residents helped us to use the entire longitudinal data obtained from the residents over the eight years. Also, using residents as random effects in model construction helped to personalize the model predictions. Therefore, even though we had a smaller population to work with, we could use thousands of measurements to build an effective model.

Personalized FH trajectories could equip healthcare providers at TigerPlace with early health risk indications and context about the changes in FH in the residents. In addition to providing a visual representation of the change in FH the model could also provide detailed information about what changes in the new assessments led to the change in FH. This could help care providers to decide on necessary targeted interventions faster.

## Conclusions

We developed and validated a model to track personalized functional health in older adults using multiple construct validators. We demonstrated that significant changes in the functional health trajectory could be early indicators of possible adverse health events. The FH trajectories could help caregivers decide appropriate interventions based on trends in overall functional health change. We propose that a larger dataset could be used in future studies to improve the model.

### Supplementary information

**Additional file 1:** Predictive ability of FHV compared to the individual health assessments.

## Figures and Tables

**Fig. 6:**
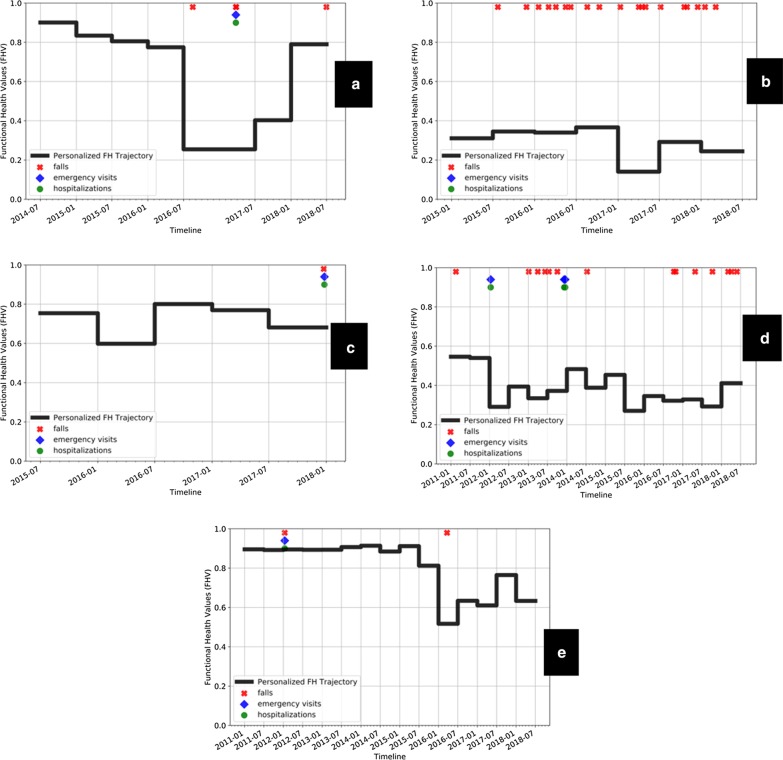
FH trajectories of TigerPlace Residents. **a** significant drop in FHV between 2016–2018 corresponds to hospitalizations and falls. **b** FHV continuously declined until Jan 2016 (~ 60) and never improved, followed by several falls. **c** An example of consistent functional health with no significant FHV declines. Several health events were observed close to death. **d** Lower FHV (< 60), corresponding to a significant number of falls and other health events throughout the stay. **e** The initial fall and other health events cannot be explained (FHI ~ 70). FHI improved over time until June 2014. Significant drops in FHI during July-Dec 2015 and January-June 2018 correspond to falls

## Data Availability

The datasets generated and analyzed during the current study are not publicly available but are available from the corresponding author on reasonable request and IRB approval.

## Appendix 1: FH trajectories of tigerplace residents

Figure 6 shows FH trajectories of five different TigerPlace residents.

## Abbreviations

EMR: Electronic medical record
FH: Functional health
FHV: Functional health values
FH trajectory: Functional health trajectory
ADL: Activities of Daily Living
IADL: Instrumental Activities of Daily Living
MMSE: Mini-Mental State Examination
GDS: Geriatric Depression Scale
SF12: Short Form 12
PCS: Physical composite score of SF12
MCS: Mental composite score of SF12
AUC: Area under the receiver operating characteristic curve
RAI MDS 2.0: Resident Assessment Instrument-Minimum Data Set, Version 2.0
