# Supplementary material for: Tracking personalized functional health in older adults using geriatric assessments
Source: BMC Med Inform Decis Mak. 2020 Oct 20;20:270. doi: 10.1186/s12911-020-01283-y (PMC7576843; doi:10.1186/s12911-020-01283-y)
Supplement: Supplementary file 1 — Additional file 1: Predictive ability of FHV compared to the individual health assessments. [file 12911_2020_1283_MOESM1_ESM.docx]

Supplementary tables

Supplementary Table 1. Predictive ability of FHV and individual health assessments. (ROC areas)

|  | Separating *no health events* category from the rest of the adverse health event categories |
| --- | --- |
|  | ROC area |
| FHV | 0.85 |
| ADL | 0.68 |
| IADL | 0.66 |
| MMSE | 0.57 |
| GDS | 0.56 |
| SF-12 MCS | 0.52 |
| SF-12 PCS | 0.63 |
